# Physicochemical Properties and Effects of Honeys on Key Biomarkers of Oxidative Stress and Cholesterol Homeostasis in HepG2 Cells

**DOI:** 10.3390/nu13010151

**Published:** 2021-01-05

**Authors:** Huong Thi Lan Nguyen, Stefan Kasapis, Nitin Mantri

**Affiliations:** 1 The Pangenomics Lab, School of Science, RMIT University, Melbourne, VIC 3083, Australia; ntlanhuong77@gmail.com (H.T.L.N.); Stefan.Kasapis@rmit.edu.au (S.K.); 2 Vietnam Institute of Agricultural Engineering and Post-Harvest Technology, Hanoi 10000, Vietnam; 3 The UWA Institute of Agriculture, The University of Western Australia, Perth, WA 6001, Australia

**Keywords:** honey, phytochemicals, antioxidants, cholesterol, oxidative stress, gene expression

## Abstract

Manuka honey and newly developed honeys (arjuna, guggul, jiaogulan and olive) were examined for their physicochemical, biochemical properties and effects on oxidative stress and cholesterol homeostasis in fatty acid-induced HepG2 cells. The honeys exhibited standard moisture content (<20%), electrical conductivity (<0.8 mS/cm), acidic pH, and monosaccharides (>60%), except olive honey (<60% total monosaccharides). They all expressed non-Newtonian behavior and 05 typical regions of the FTIR spectra as those of natural ones. Guggul and arjuna, manuka honeys showed the highest phenolic contents, correlating with their significant antioxidant activities. Arjuna, guggul and manuka honeys demonstrated the agreement of total cholesterol reduction and the transcriptional levels of *AMPK*, *SREBP2*, *HCMGR*, *LDLR*, *LXRα*. Jiaogulan honey showed the least antioxidant content and activity, but it was the most cytotoxic. Both jiaogulan and olive honeys modulated the tested gene in the pattern that should lead to a lower TC content, but this reduction did not occur after 24 h. All 2% concentrations of tested honeys elicited a clearer effect on *NQO1* gene expression. In conclusion, the new honeys complied with international norms for natural honeys and we provide partial evidence for the protective effects of manuka, arjuna and guggul honeys amongst the tested ones on key biomarkers of oxidative stress and cholesterol homeostasis, pending further studies to better understand their modes of action.

## 1. Introduction

Atherosclerosis is a leading cause of mortality worldwide and its pathogenesis is associated with several risk factors, including oxidative stress and hypercholesterolemia [1]. As most of plasma cholesterol is low density lipoprotein cholesterol (LDL-C) which forms plaque and hardens the arteries at elevated levels, excessive LDL-C is a detrimental element that exacerbates the severity of cardiovascular diseases [2,3]. Statin drugs are extensively used to treat hypercholesterolemia due to their ability of inhibiting hydroxy methylglutaryl coenzyme A reductase (HMGCR), a rate-limiting enzyme in de novo cholesterol synthesis. However, it was reported that a healthy lifestyle and dietary plan significantly decrease cholesterol levels, thus, many people on the brink of abnormal cholesterol level can return to normal cholesterol status without medications [4].

Honey has attracted great attention as a natural sweetener that promotes health because it contains a high quantity of reducing sugars, a noticeable content of enzymes, proteins, vitamins and minerals and a wide range of phytochemicals. Due to the diversity of plant metabolites in floral nectars, chemical profiles and consequently, antioxidant activities of honeys are diverse [5,6,7]. The oxygen radical absorbance capacity of honey (3–17 µmol TE/g) is comparable to that of several fresh fruit and vegetables (0.5–19 µmol TE/g) [8]. The benefits of honey on cholesterol homeostasis have been well documented [9]. Particularly, phenolic compounds such as catechin, quercetin, luteolin in honey are reported to improve coronary vasodilation, prevent blood clots and LDL-C oxidation [10,11,12,13]. Several flavonoids in honey are in aglycon forms, making them readily bioavailable when consumed [14]. However, to date, the understanding of the molecular mechanism through which honey elicits its protection against oxidative stress and hypercholesterolemia is limited.

It has been reported that oxidative stress closely links to dysregulated lipid homeostasis, thus, we explored the transcription factor NF-E2-related factors 2 (*Nrf2*) and its downstream gene, NAD(P)H:quinone oxidoreductase 1 (*NQO1*), which are central in defense responses to oxidative stress [15]. In addition, AMP-activated protein kinase (*AMPK*), a key energy sensor has been extensively investigated in lipid metabolism pathways because its activation induces ATP-generating pathways, including the uptake and oxidation of glucose and fatty acids [16].

Cholesterol metabolism is co-regulated by sterol regulatory element-binding protein (*SREBP-2*) and liver X receptors (*LXRs*) in the liver. *SREBP*-2 preferentially modulates genes associated with cholesterol synthesis and uptake (*HMGCR* and *LDLR*, respectively) [17]. *LXRs* are responsible for reducing cholesterol absorption in intestine and promoting the excretion of bile cholesterol in the liver through upregulating membrane transporters [18], whereas proliferator-activated receptor alpha (*PPARα*) is a critical molecule that controls the transcription of downstream genes of the lipid catabolism. The activation of *PPARα* enhances the β-oxidation of fatty acids, reducing the content of cellular lipids [19]. As such, in the current study, manuka honey and four other honeys newly developed in The Pangenomics Laboratory, RMIT University, were examined for their standard quality and effects on the aforementioned biomarkers in fatty acid-induced HepG2 cells. This cell line was used as it is highly differentiated and exhibits several genotypic characteristics of normal liver cells, including cholesterol synthesis and excretion [20].

## 2. Materials and Methods

### 2.1. Honey Samples

A honey analogue (40% fructose, 35% glucose, 5% sucrose, *w*/*w*) was prepared to contrast the effects of honeys. A commercial manuka honey containing methylglyoxal 400+ mg/kg (manuka) and four medicinal honeys developed from the extracts of *Terminalia arjuna* bark (aruna honey), *Commiphora mukul* stem (guggul honey), *Gynostemma pentaphyllum* leaves (jiaogulan honey) and *Olea europaea* fruit retentate (olive honey) were included in the study.

Four healthy bee colonies provided by Berley Honey were kept separately in semi-control enclosures and each was fed with a medicinal nectar, which is a solution of sugars, amino acids and a medicinal plant extract (Table 1). Briefly, sugar proportion was prepared following the sugar pattern of *melitophilus* flowering plant species, which are beneficial for honeybees [21,22]. A range of amino acids was prepared following the profile of amino acids identified in flower nectar of *Prunella vulgaris,* which is a bee-friendly herb species [23,24]. The inclusion of plant extract was adjusted to the bees’ preference to the nectar. In addition, pollen granules A^+^ premium grade (Western Australia) was given daily as a protein source to maintain bee colonies. Extracts of *T. arjuna* bark, *C. mukul* stem and *G. pentaphyllum* leaves were obtained following methods described by Sultana et al. [25], Jasmine et al. [26] and Liu et al. [27] with some minor modifications. Olive retentate (Boundary Bend, VIC, Australia) was directly used to replace plant extracts in the preparation of nectar as it is a liquid that contains (mg/100 mL): total phenol content 90.5, hydroxytyrosol 15, sodium 1.9, total sugars < 1.9, fat < 0.2, protein 0.1.

### 2.2. Moisture, pH, Electrical Conductivity

Moisture, pH and electrical conductivity were measured following the International Honey Commission method [29]. Moisture content was recorded using a Refracto 30GS refractometer (Mettler Toledo, Port Melbourne, VIC, Australia) and converted following a Chetaway table. pH value was measured from 10% honey solutions. Electrical conductivity was measured from 20% honey solutions (*w*/*v*) using a S230 conductivity meter (Mettler Toledo, Port Melbourne, Australia).

### 2.3. Reducing Sugars

Reducing sugars, D-glucose and D-fructose, in honeys were analyzed following instructions from K-SUFRG 06/14 kit (Megazyme, Melbourne, VIC, Australia). Briefly, D-glucose was assayed by utilizing hexokinase and glucose-6-phosphate avoiding sucrose hydrolysis. The content of D-fructose was recorded after the analysis of D-glucose content, following isomerization with phosphoglucose isomerase.

### 2.4. Color Intensity

Color intensity of honeys was measured following Saxena, Gautam and Sharma [30]. Honey solutions (50%, *w*/*v* in Milli-Q water) were filtered using 0.45 µm sterile Millex filters (Merk, Bayswater, VIC, Australia) to remove particles. Results were calculated as the difference of the 450 and 720 nm absorbance readings recorded from a Lambda 35 UV–vis spectrophotometer (Perkin Elmer, Waltham, MA, USA).

### 2.5. Protein Content

Protein content was assayed following Bradford’s method (1976) using a Thermo Scientific™Coomassie Protein Assay Kit (Thermo Fisher, Scoresby, VIC, Australia). In doing so, a volume of 20 µL of honey solution (10%, *w*/*v*) was mixed with 250 μL of Coomassie reagent in a 96-well microplate. After ten min incubation at room temperature, the plate was read at 595 nm absorbance using a POLARstar Omega plate reader (BMG Labtech, Ortenberg, Germany). A calibration curve using bovine serum albumin (BSA) standards in the range of 0 to 100 μg/mL was used to calculate the protein content.

### 2.6. Total Phenolic Content

Total phenolic content was assayed using Folin–Ciocalteu reagent [31]. Briefly, 0.5 mL of 10% filtered honey solution (*w*/*v*) was mixed with 2.5 mL of 0.2 N Folin–Ciocalteu reagent (Sigma-Aldrich, Castle Hill, NSW, Australia). Subsequently, 2 mL of 75 g/L Na_2_CO_3_ solution was added. After 2 h incubation at ambient temperature, the mixture was read at 760 nm absorbance using a Lambda 35 UV–vis spectrophotometer (Perkin Elmer, Waltham, MA, USA). Data are the mean of four replicates ± SD. Gallic acid standards ranging from 0 to 100 μg/mL were used to construct a calibration curve.

### 2.7. Total Flavonoid Content

Total flavonoid content was measured following the method described by Islam et al. [32]. Two grams of honey was diluted into 10 mL methanol and filtered with a 0.45 µm sterile Millex filter (Merk, Bayswater, VIC, Australia). Then, 1 mL of the 20% honey solution (*w*/*v*) was added to 4 mL milli-Q water, followed by 0.3 mL of 5% NaNO_2_ (*w*/*v*). After five min, 0.3 mL of 10% AlCl_3_ (*w*/*v*) was added. Six min later, the mixture was neutralized with 2 mL of 1.0 mM NaOH solution and increased into 10 mL using water. The absorbance was recorded at 510 nm within 5 min using a Lambda 35 UV–vis spectrophotometer (Perkin Elmer, Waltham, MA, USA). Catechin standards ranging from 0 to 100 μg/mL were used to construct a calibration curve against a blank consisted of honey solution and methanol without AlCl_3_.

### 2.8. Radical Scavenging Activity and Ascorbic Acid Equivalent Antioxidant Content

Radical scavenging activity (RSA) of the medicinal honeys was recorded by determining the reduction in radical 2,2-diphenyl-1-picrylhydrazyl (DPPH) following Meda et al. [33]. Honey was diluted in methanol and filtered with a 0.45 µm sterile Millex filter (Merk, Bayswater, VIC, Australia). Then, 0.8 mL of a 2.5% honey solution (*w*/*v*) was added to 2.7 mL of 0.024 mg/mL DPPH in methanol. The mixture was vigorously shaken for 15 s and incubated for 30 min without light at room temperature. The DPPH reduction was read at 517 nm absorbance using a Lambda 35 UV–vis spectrophotometer (Perkin Elmer, Waltham, MA, USA). RSA was the percentage of DPPH discoloration calculated from the equation:

% RSA = [(ADPPH − AS)/ADPPH] × 100(1)

where ADPPH and AS are the absorbance units of DPPH solutions in the absence and presence of honey, respectively.

The antioxidant content (AEAC) was analyzed as aforementioned and referred to calibration curve of ascorbic acid standards ranging from 0 to 15.0 μg/mL.

### 2.9. Viscosity

Viscosity was measured using an AR-G2 rotational rheometer (TA instruments, New Castle, DE USA). Honeys (1.5 g) were loaded onto a 40 mm-diameter parallel plate geometry at room temperature and the analysis was set at 500 µm gap and shear rate from 0.01 to 10 cm^−1^ at 20 °C.

### 2.10. Fourier-Transform Infrared Spectroscopy (FTIR)

FTIR spectra of the medicinal honeys were scanned using A MIRacleTM ZnSe single reflection ATR plate-linked spectrometer (Perkin Elmer, Norwalk, CT, USA). An amount of 0.2 g of honeys was placed onto the measuring plate and measured for 40 scans from 4000 to 500 cm^−1^, 4 cm^−1^ resolution at room temperature.

### 2.11. Cell Culture

Human liver cell line (HepG2) was kindly granted by School of Health and Biomedical Sciences, RMIT University. HepG2 cells were routinely maintained in a complete Dulbecco’s modified Eagle’s medium (DMEM) containing fetal bovine serum (FBS, 10%, *v*/*v*) (Thermo Fisher, Scoresby, VIC, Australia), glutamine (2 mmol/L), penicillin (100 U/mL), and streptomycin (100 mg/mL) in an incubator set at 37 °C and 5% CO_2_ atmosphere. The complete medium was changed twice a week and subcultures were performed when the cells reached a 75% confluence. Cells were counted using trypan blue reagent and Countess^®^ Automated Cell Counter (Thermo Fisher, Scoresby, VIC, Australia). All assays used the cells at passage numbers less than twenty.

### 2.12. Cell Viability

The viability of HepG2 cells under the effect of medicinal honeys was assayed using PrestoBlue^®^ kit (Invitrogen, Australia). An amount of 100 μL of complete DMEM medium containing 3 × 10^4^ cells/well was pipetted on a 96-well plate and incubated at 37 °C and 5% CO_2_. Ten hours later, the media was discarded, and the cells were incubated in free-serum DMEM overnight. Honey solutions in a range of 0–20% (*w*/*v*) were prepared in 2% FBS-DMEM and applied to the cells. After 24-, 48- and 72-h incubation with honeys, the cells were washed with PBS twice followed by adding PrestoBlue reagent 10.0% in free-serum medium followed by another two-hour incubation. The fluorescence intensity was recorded at 535 nm/590 nm using a POLARstar spectrophotometer (BMG LabTech, Ortenberg, Germany). Cell viability was calculated as the percentage of the fluorescence unit of treated cells to control, which is untreated cells.

### 2.13. Cell Treatment with Fatty Acids

HepG2 cells (6 × 10^5^ cells/well) in complete DMEM medium were seeded on a 6-well plate. Ten hours later, the complete medium was discarded, and the cells were maintained in serum-free, high glucose DMEM medium overnight. A conjugation of fatty acids 1.0 mM (oleic acid/palmitic acid, 2:1) and fat-free BSA (Sigma-Aldrich, Castle Hill, NSW, Australia) was prepared at a 6:1 ratio in 2.0% FBS-DMEM medium and delivered to the cells. In addition, honey was simultaneously added at final concentrations 1.0% and 2.0% (*w*/*v*). Control cells were exposed to 1.0% BSA (*w*/*v*). Cell pellets were collected after 24 h.

### 2.14. Analysis of Total Cellular Cholesterol and Triglyceride Content

Lipids were extracted from cell pellets following Folch et al. [34]. The dry lipid was dissolved in 200 μL methanol for the subsequent determination of total cellular cholesterol (TC) and triglycerides (TG) by enzymatic methods, using Amplex Red Cholesterol Assay Kit (Thermo Fisher, Scoresby, VIC, Australia) and EnzyChrom Triglyceride Assay Kit (BioAssay Systems, Hayward, CA, USA) respectively. Cellular protein content was assayed based on bicinchoninic acid (BCA) method using BCA Protein Assay Kit (Thermo Fisher, Scoresby, VIC, Australia). TC and TG contents were normalized to relevant protein content (µg/mg cellular protein) and expressed in fold change relative to control (1.0 mM fatty acid treatment).

### 2.15. Real-Time Quantitative Polymerase Chain Reaction (RT qPCR)

Total cellular RNA was isolated from the HepG2 cells using RNeasy mini kit (Qiagen, Chadstone, VIC, Australia). Complementary DNA templates were synthesized from 500 ng of total RNA in 20 μL assays, using SensiFAST cDNA Synthesis kit (Bioline). Reactions were set at 25 °C for 10 min, followed by 42 °C for 15 min, 48 °C for 15 min, 85 °C for 5 min and finally cooled at 4 °C before being stored at −20 °C. Primers (Table 2) were obtained from Bioneer Pacific (Kew East, VIC, Australia).

RT qPCR analyses were set up as described in SensiFAST™ SYBR No-ROX kit (Bioline, Eveleigh, NSW, Australia) and performed at 95 °C for 2 min, succeeded by 40 cycles of 95 °C for 5 s, 60 °C for 10 s, 72 °C for 10 s using a Rotor-Gene Q machine (Qiagen, Chadstone, VIC, Australia). β-actin was used as a reference gene. The primer specificity was confirmed by the analysis of melting curve. The relative quantification of gene expression was accomplished following 2^−ΔΔCT^ method [35]. Primer sequences are presented in the supplementary part (Table S1).

### 2.16. Statistical Analysis

Data were analyzed using Excel (Office 365), IBM SPSS Statistics 26 software and expressed as mean of four replicates ± standard deviation (SD) (SPSS Inc., Chicago, IL, USA). Statistical analysis was performed by using one-way analysis of variance (ANOVA), followed by Duncan’s multiple comparison test. Results were considered statistically different when *p* ≤ 0.05.

## 3. Results and Discussion

### 3.1. Physicochemical Properties

#### 3.1.1. Moisture, Electrical Conductivity, pH and Color Intensity

Moisture content (MC) is a critical factor determining honey quality and affecting other physical properties of honey such as sugars, crystallization, viscosity and stability [36]. High MC also promotes the growth of osmotolerant yeasts, which deteriorates the quality of honey. MC depends on several factors such as weather and maturity of honey. In the present study, moisture content was considerably low in arjuna, guggul and jiaogulan honeys (14.9%, 13.4% and 14.0%, respectively), compared to manuka and olive honeys (18.5% and 18.4%) (Table 2). The results indicated MC values recorded in the tested honeys (including the 4 we produced) were well below 20.0%, the imposed limit for natural nectar honeys and consistent to previous findings which reported a moisture range of 13.0–19.0% for natural honeys from various origins [36,37].

Electrical conductivity (EC) indicates the quantity of mineral elements, organic acids and proteins in the composition of honey. EC recorded in all medicinal honeys was less than 0.8 mS/cm, the maximal limit regulated for blossom honey and was comparable to the other findings [37,38]. We also found that EC values in the newly developed medicinal honeys (0.24–0.34 mS/cm) were lower than those in manuka honey (0.54 mS/cm). This could result from the variations in nectar composition and environment that honeybees are exposed to [39].

pH value of honey is another important feature affecting its texture, stability and shelf-life. pH of all tested honeys was in a limited range of 3.9–4.2 (Table 2). The results agree with earlier studies reporting acidic pHs ranging from 3.7 to 4.5 for different natural honeys [39,40].

Color intensity (ABS_450_) is proportional to the content of pigments (carotenoids, flavonoids) and antioxidant activity of honey [8]. Color intensity of the medicinal honeys greatly varied, with the highest value noted for manuka honey (2201.1 mAU), whereas much lower values were recorded for the four new medicinal honeys (462.3, 722.7, 171.7 and 243.5 mAU for arjuna, guggul, jiaogulan and olive honey, respectively). The findings were in line with other observations, in which color intensity of natural honey ranges from 125.0 to 3400.0 mAU [41,42,43].

#### 3.1.2. Viscosity

**Figure 1:**
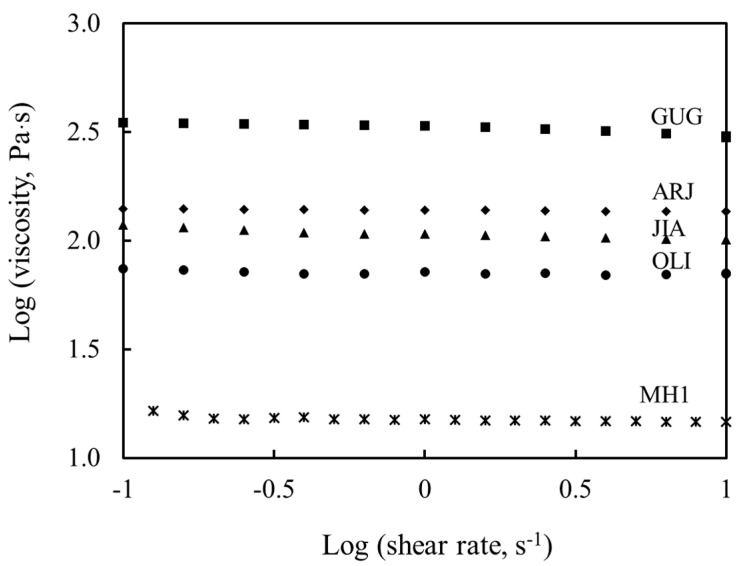
Viscosity and rheological behavior of the tested honeys. MH1: manuka honey containing methylglyoxal 400^+^ mg/kg, ARJ: arjuna honey, GUG: guggul honey, JIA: jiaogulan honey and OLI: olive honey. The viscosity of all the tested honeys remained constant with the increase in experimental shear rate, indicating their Newtonian liquid behavior.

The viscosity data indicated all tested honeys were Newtonian liquids (Figure 1), which is consistent with most natural honeys [44,45]. The novel medicinal honeys demonstrated much higher viscosity than manuka honey. Guggul honey presented the highest viscosity (316.2 Pa·s), followed by arjuna, jiaogulan and olive honey (125.8, 100.0 and 63.1 Pa·s). Although manuka honey showed the lowest viscosity (15.8 Pa·s), its viscosity is comparable with that of other natural honeys (1.8–13.8 Pa·s) [46]. In addition, the viscosity data were inversely correlated with moisture contents of the relevant honey types (Table 2), congruent with previous findings [30,45].

#### 3.1.3. Fourier-Transformed Infrared Spectroscopy

**Figure 2:**
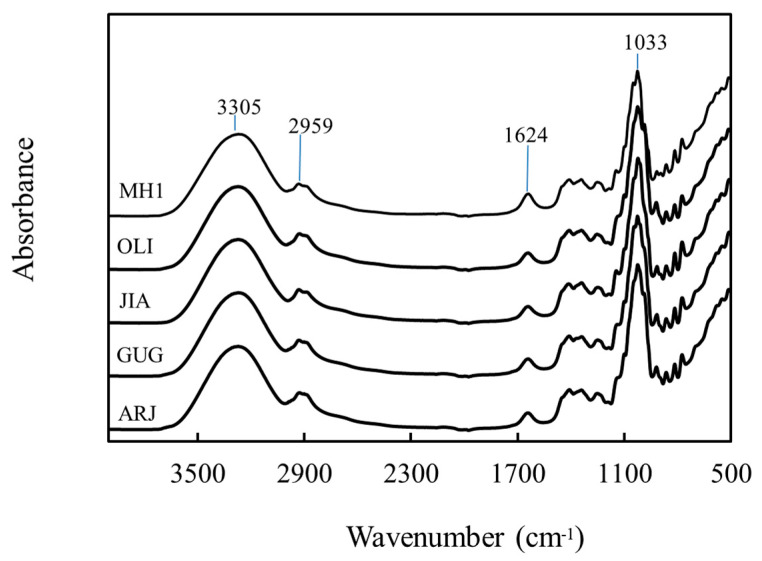
Chemometric analysis of honeys using Fourier-transform infrared spectroscopy (FTIR) scanned from 4000 to 400 cm^−1^. MH1: manuka honey containing methylglyoxal 400 mg/kg, ARJ: arjuna honey, GUG: guggul honey, JIA: jiaogulan honey and OLI: olive honey. The values indicate peaks of vibrational regions representing functional groups typical for natural honeys. The regions of 3700–3000 cm^−1^ and 3000–2800 cm^−1^ indicate O-H stretching of water molecules, and C-H stretching of sugars, respectively. A peak at 1624 cm^−1^ in all honeys is assigned for the bending of H-O-H molecules. The 1540–1175 cm^−1^ region indicated bending modes for C-O-H, C-C-H, and O-C-H groups. The 1175–940 cm^−1^ fingerprint region was specific for stretching modes of C-O and C-C of carbohydrates, and bands in 900–750 cm^−1^ indicated the stretching modes of C-O and C-C of saccharide molecules.

Chemometric analysis of the medicinal honeys using Fourier-transform infrared spectroscopy (FTIR) exhibited five vibrational regions assigned for specific chemical bonds that are typical for natural honeys (Figure 2). The regions of 3700–3000 cm^−1^ and 3000–2800 cm^−1^ indicate the O–H stretching of water molecules, and C–H stretching of sugars, respectively [47]. A peak appears at 1624 cm^−1^ in all honeys and is assigned for the bending of H–O–H molecules [48]. The 1540–1175 cm^−1^ region indicated the presence of bending modes for C–O–H, C–C–H, and O–C–H groups [49]. The vibrations with strong intensity absorbance in 1175–940 cm^−1^ bands known as a fingerprint region for honey’s sugars were specific for stretching modes of C–O and C–C from carbohydrates (Anjos, Campos, Ruiz, and Antunes, 2015). Bands in the range 900–750 cm^−1^ indicated the stretching modes of C–O and C–C of saccharide molecules [9,43]. The results indicate a similarity in typical functional groups of manuka honey and the four new honeys.

### 3.2. Biochemical Characteristics

#### 3.2.1. Reducing Sugars

Monosaccharides (fructose and glucose) are the major components of honey and they govern the physical state of honey. Table 3 indicates that the total content of fructose and glucose is over 60 g/100 g for all medicinal honeys, except olive honey (49.2 g/100 g honey). The results comply with the regulation by Council Directive 2001/110/EC [37] and similar with other natural honeys having total monosaccharides within the range of 43–75 g/100 g honey [30,50]. In addition, the ratio of fructose/glucose (F/G) is important for the development of crystal sugar nuclei in honey, as it has been reported that honey having F/G ratio > 1 can be stored longer without crystallization, in contrast, honey having F/G ratio < 1 develops crystals rapidly, leading to unfavored color and texture. In the current study, all tested honeys presented F/G ratios > 1 that are comparable to most natural honeys [43,50].

#### 3.2.2. Protein Content

Protein content is determined by the presence of amino acids, nectar sources, enzymes secreted from honey bees and storage conditions [30]. Manuka honey exhibited the highest protein content (128.5 mg/100 g) as this honey is categorized as “high protein” compared to other natural commercial honeys [43]. The four other honeys contain lower protein contents than manuka, varying from 111.4 for jiaogulan honey to 66.6, 55.3 and 25.6 (mg/100 g) for olive, guggul, and arjuna honey, respectively.

#### 3.2.3. Total Phenolic and Flavonoid Content

Phenolic and flavonoid compounds are markers of antioxidant activity and the content of total phenolics (TPC) and flavonoids (TFC) were commonly analyzed to predict therapeutic potential of natural products [14,28]. Guggul honey contained the highest level of phenolic compounds (108.2 mg GAE/100 g), followed by manuka and arjuna honeys (72.1 and 73.4 mg GAE/100 g), whereas olive honey contained a moderate level (59.8 mg/100 g) and jiaogulan honey showed the least quantity (31.6 mg/100 g) (Table 3). The data are comparable with those recorded in other natural honeys, ranging from 16.02 to 120.04 mg GAE/100 g [28,40] and are supportive of the color intensity data, meaning honey with higher color values has more phenolic content [51]. We also found a strong correlation of TPC and color intensity (R^2^ = 0.930) for all four new honeys but not for manuka honey.

Total flavonoid contents (TFC) greatly varied among the honeys. Although manuka honey contained a higher TFC (3.9 mg/100 g) than many natural honeys (0.47–3.61 and 2.0–5.4 mg/100 g honey) [40,52], its TFC was similar to guggul honey (4.1 mg/100 g) and much lower than that of arjuna and olive honeys (15.3 and 24.0 mg/100 g, respectively), whereas jiaogulan honey had the lowest TFC (2.1 mg/100 g). The results confirmed TPC and TFC of honeys vary and they are strongly determined by nectar sources.

#### 3.2.4. Radical Scavenging Activity and Ascorbic Acid Equivalent Antioxidant Content

Radical scavenging activity (RSA) is extensively examined in biological samples using 2,2-diphenyl-1-picrylhydrazyl (DPPH), a stable nitrogen-centered radical. The RSA of the tested honey varied. Arjuna and guggul honeys showed the highest RSA (90.8% and 91.7%), followed by olive and manuka honeys (75.2% and 55.5%) and jiaogulan honey (21.7%). The RSA of arjuna and guggul are outstanding, while those for other tested honeys are comparable to Bangladesh and Indian natural honeys (33.6–97.5% and 44.0–71.0%, respectively) [30,32].

Ascorbic acid equivalent antioxidant content (AEAC), calculated using the calibration curve of ascorbic acid standards (R^2^ = 0.998), revealed the antioxidant potential of the medicinal honeys. The highest AEAC was for arjuna and guggul honeys (81.0 and 81.9 mg/100 g, respectively), followed by olive and manuka honeys (67.1 and 49.6 mg/100 g, respectively) and the lowest was found in jiaogulan honey (19.4 mg/100 g). Except jiaogulan honey, the tested honeys showed higher AEAC values compared to Burkina Fasan, India and Bangladesh honeys (10.20–37.87 mg AEAC/100 g) [30,32,33]. The AEAC data exhibited a good agreement with RSA in all tested honeys, confirming the outstanding antioxidant content and activity of arjuna and guggul honeys, followed by olive honeys.

### 3.3. Effect of Honey on the Viability of HepG2 Cells

**Figure 3:**
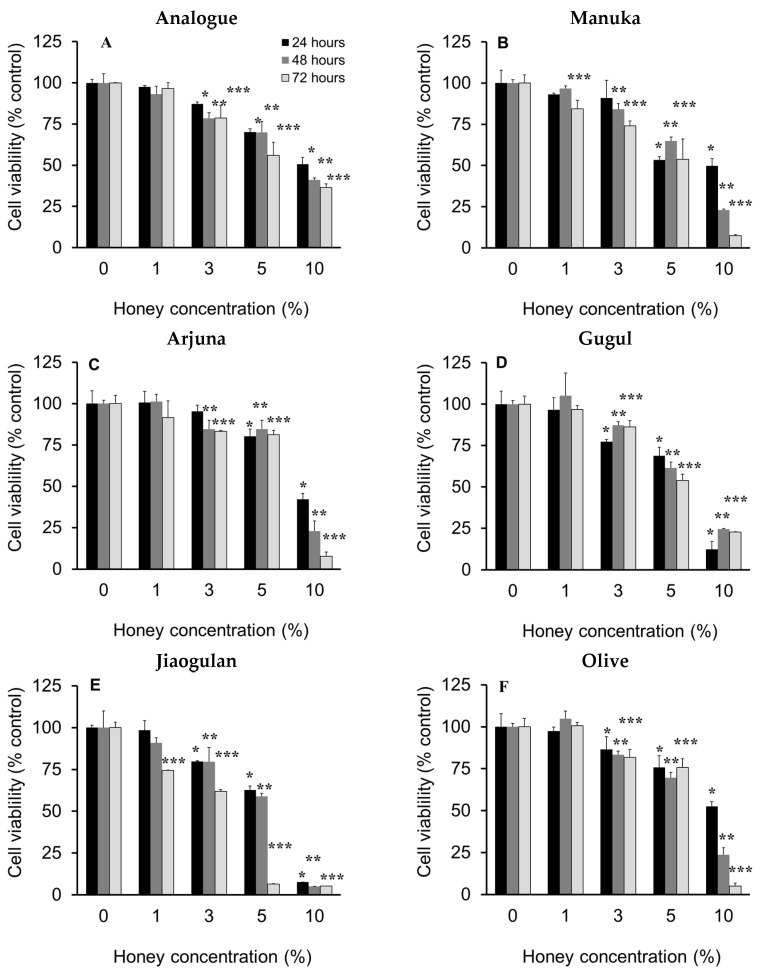
The viability of HepG2 cells exposed to the tested honeys for 24 h (black), 48 h (grey) and 72 h (whitist) incubation. Results are the mean ± SD of four replicates. *, **, *** signifies *p* ≤ 0.05 vs. control (0% honey) after 24 h, 48 h, and 72 h incubation, respectively, analyzed by one-way ANOVA followed by Duncan’s multiple comparison test. (**A**): Analogue, (**B**): manuka; (**C**): arjuna, (**D**): guggul, (**E**): jiaogulan, (**F**): olive honey.

Cells exposed to biologically toxic materials may change their morphology, growth and death rate, thus disintegration may occur. Therefore, examination of the cytotoxicity of novel drugs is crucial prior to investigations [53]. In this study, the tested honeys affected the viability of HepG2 cells in a time- and dose-dependent manner (Figure 3).

The tested honeys at 1.0% (*w*/*v*) did not affect the cell viability in the whole period of the experiment, except manuka and jiaogulan honeys which reduced cell viability to 84.4% and 74.4% after 72 h, respectively. Interestingly, the analogue, guggul, jiaogulan and olive honeys at 3.0% significantly reduced cell viability after 24, 48 and 72 h, suggest that the analogue and these honeys induced cytotoxicity in a similar pattern. Manuka and arjuna honeys at 3.0% maintained all cell viability after 24 h, and then reduced their viability after 48 and 72 h. All tested honeys at 5.0% and 10.0% significantly reduced cell viability. This could be due to the high sugar levels that induced apoptosis, which was also observed in other mammalian cell lines, such as human umbilical vein endothelial cells and HT22 hippocampal neuronal cells [54,55,56]. Noticeably, 5% jiaogulan honey reduced 93.5% cell viability after 72 h and 10% of this honey eliminated 92% of viable cells after 24 h until 72 h. The data indicated jiaogulan honey is the most cytotoxic of the tested honeys and are supportive of previous findings that reported the cancer-preventative effect of jiaogulan extracts [57].

The effect of the tested honeys on the viability of HepG2 cells are comparable with that of sugar syrup, thyme and manuka honeys on the viability of PC3, DU145 human prostate cancer cell lines, and THP-1 cell lines [58,59,60]. Taken together, honey treatments of 1.0% and 2.0% (*w*/*v*) for 24 h were selected for subsequent investigations.

### 3.4. Effect of Medicinal Honeys on the Expression of Key Genes Associated with Antioxidant Defense Responses

**Figure 4:**
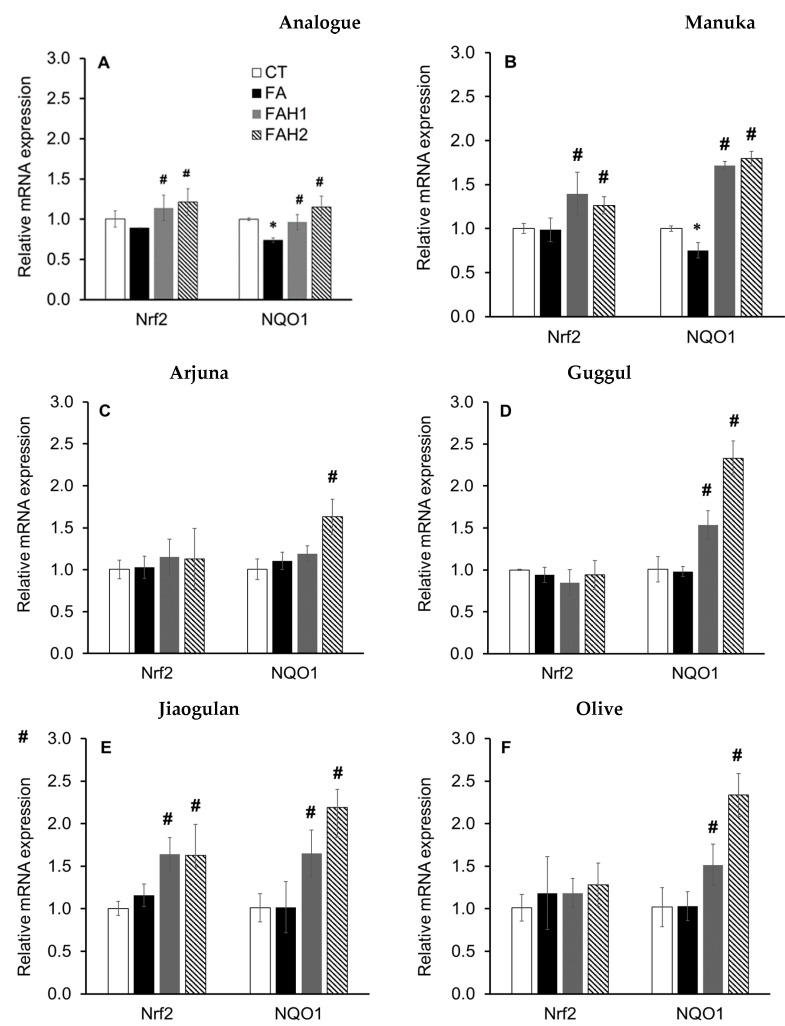
The expression of genes associated with antioxidant defense response in fatty acid-induced HepG2 cells incubated with the tested honeys after 24 h. Results are mean ± SD of four replicates; *: *p* < 0.05 vs. control and #: *p* < 0.05 vs. fatty acid-treated cells, analyzed by one-way ANOVA followed by Duncan’s multiple comparison test. (**A**): Analogue, (**B**): manuka; (**C**): arjuna, (**D**): guggul, (**E**): jiaogulan, (**F**): olive honey. CT: Control without fatty acid and honey, FA: 1 mM fatty acid treatment, FAH1: 1 mM fatty acid and 1.0% honey; FAH2: 1 mM fatty acid and 2.0% honey (*w*/*v*).

*Nrf2* and *NQO1* genes have been extensively studied for their defense responses against cytotoxicity and oxidation. Figure 4 showed that the analogue, manuka honey and jiaogulan activated *Nrf2* gene at both 1.0% and 2.0%. Other honeys did not show their effect on this gene but they upregulated the downstream *NQO1*, thus *Nrf2* had possibly been expressed differently in these honey treatments before the experimental terminating time (24 h) and later was recorded at the detected level [61].

All the tested samples upregulated NQO1 gene, but the analogue showed a lower level of transcription (<1.5 fold) compared to the honeys (>1.5 fold), suggesting an additional effect of non-sugar components of honeys on the gene. The four newly developed honeys demonstrated a stronger effect at 2.0% compared to 1.0% on the increase in *NQO1* mRNA abundance (Figure 4C–F).

This observation is supported by the high contents of phenolics/flavonoids, RSA and AEAC recorded in arjuna, guggul and olive honeys (Table 3) and agrees with previous investigations reporting that polyphenols modulate Kelch-like ECH associated protein 1/Nrf2/antioxidant response elements (Keap1/Nrf2/ARE) gene pathway that leads to the amelioration of oxidative stress in vitro and in vivo [15,61,62,63].

### 3.5. Effect of Medicinal Honeys on Cellular Lipid Content

**Figure 5:**
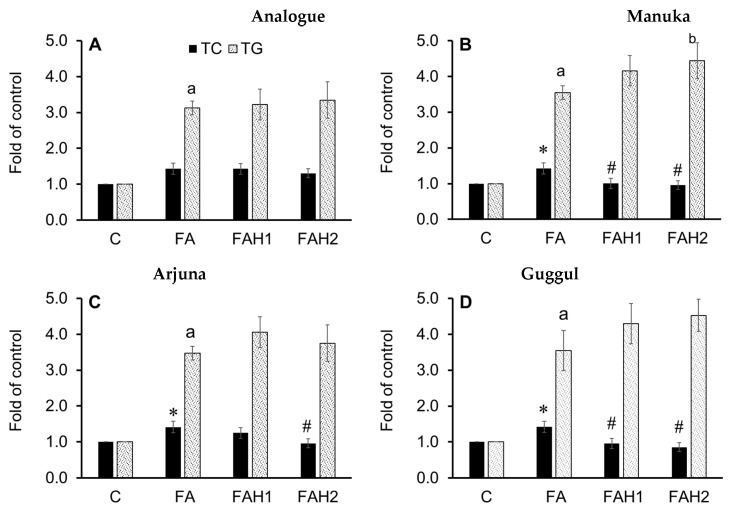

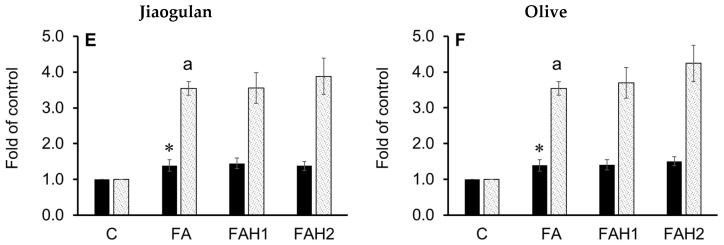
Total cellular cholesterol (TC) and triglyceride (TG) content in fatty acid-induced HepG2 cells incubated with the tested honeys after 24 h. C: Control cells, FA: fatty acid treatment, FAH1: fatty acids and honey 1.0%, FAH2: fatty acids and honey 2.0%. Results are mean ± SD of four replicates; *: *p* < 0.05 vs. control and #: *p* < 0.05 vs. fatty acid-treated cells for TC; a: *p* < 0.05 vs. control and b: *p* < 0.05 vs. fatty acid-treated cells for TG. Data were analyzed by one-way ANOVA followed by Duncan’s multiple comparison test. (**A**): Analogue, (**B**): manuka; (**C**): arjuna, (**D**): guggul, (**E**): jiaogulan, (**F**): olive honey.

HepG2 cells were treated with individual honeys (1.0% and 2.0%) in the presence of 1.0 mM of fatty acids (oleic/palmitic; 2:1) and their total cellular cholesterol (TC) and triglycerides content (TG) were recorded after 24 h (Figure 5). TC in fatty acids treatment increased, but it reduced when adding manuka and guggul (1.0% and 2.0%) and arjuna (2.0%) (Figure 5B–D). However, TC did not drop when the cells were treated with the analogue, jiaogulan and olive honey (Figure 5A,E,F). Cellular TG strongly increased (>3.0 fold of the control) by fatty acids treatments and the addition of any medicinal honey did not reduce TG accumulation in the liver cells. The results will be discussed together with gene expression data.

### 3.6. Effect of Medicinal Honeys on Key Biomarkers of Cholesterol Homeostasis

**Figure 6:**
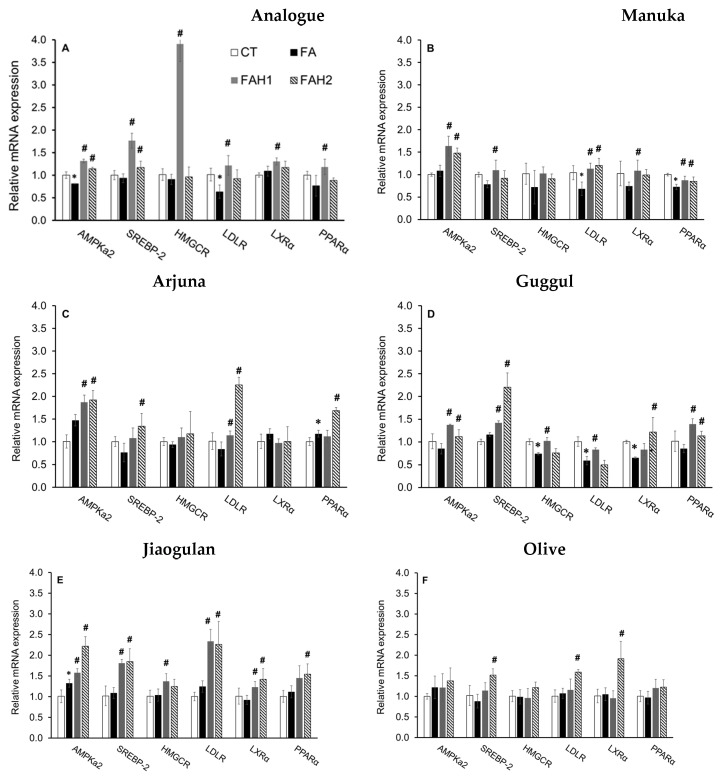
The expression of genes associated with cholesterol homeostasis in fatty acid-induced HepG2 cells incubated with the tested honeys after 24 h. Results are mean ± SD of four replicates; *: *p* < 0.05 vs. control and #: *p* < 0.05 vs. fatty acid-treated cells, analyzed by one-way ANOVA followed by Duncan’s multiple comparison test. (**A**): Analogue, (**B**): manuka; (**C**): arjuna, (**D**): guggul, (**E**): jiaogulan, (**F**): olive honey. CT: Control without fatty acid and honey, FA: 1 mM fatty acid treatment, FAH1: 1 mM fatty acid and 1.0% honey; FAH2: 1 mM fatty acid and 2.0% honey (*w*/*v*).

To understand the effect of honey on cholesterol homeostasis at molecular levels, we explored the expression of associated genes in HepG2 cells (*AMPKα*, *SREBP*-2, *HMGCR*, *LDLR*, *LXRα* and *PPARα*) using RT qPCR analysis (Figure 6). As honey is composed of sugar and non-sugar components, an analogue which consists of sugars similar to the composition of honeys (40% fructose, 35% glucose and 5% sucrose) was examined for the effect of the sugars on the genes and relative contrast to the tested honeys.

The analogue (Figure 6A) at 1.0% and 2.0% stimulated *SREBP2* through the activation of *AMPKα.* It also upregulated *SREBP2* downstream genes, *HMGCR* and *LDLR,* at 1.0% but not at 2.0% (3.9- and 1.2-fold change relative to control, respectively). This finding is consistent with an increased TC content (Figure 5A) and is in line with previous studies [64,65], suggesting that 1.0% analogue stimulates the cholesterol synthesis in the fatty acid-induced HepG2 cells, likely through the *SCAP-SREBP2* pathway by lowering free cholesterol in the cells and/or desensitizing SCAP to cholesterol [64]. The 1% analogue also upregulates the *LXRα* and *PPARα* (Figure 6A). The results are consistent with a recent study reporting either glucose alone (25 mM) or a mixture of glucose and fructose upregulated *LXRα* in the presence of 1.0 mM fatty acids [65]. As *LXRs* function in cholesterol absorption, transport, efflux, and excretion process, their upregulation improves reverse cholesterol transport and circulation of high-density-lipoprotein [66]. Interestingly, 2% analogue did not show any effect on the transcript level of the target genes, although a significant increase in TC was recorded. It has been extensively known that *SREBP2* works in a negative feedback mechanism, which means an increase in cellular cholesterols suppresses *SREBP2* and downregulates its target genes [67]. Thus, the elevation of *SREBP2* mRNA abundance at 2.0% analogue could result from the activation of *AMPK* but then the transcription of its downstream genes possibly was suppressed by the increased accumulation of TC (Figure 5A and Figure 6A).

The tested honeys (excluding olive honey) at both 1.0% and 2.0% (*w*/*v*) activated *AMPKα* but the expression levels of this gene varied, depending on honey type and concentration (Figure 6). In addition, all tested honeys at 1.0% induced *HMGCR* gene expression to the level close to the control, indicating that the honeys modulated *HMGCR* gene expression against the analogue (3.9-fold change compared to control).

Manuka honey (Figure 6B) at 1.0% and 2.0% maintained *HMGCR* mRNA abundance as in the control, but it upregulated *LDLR* gene expression. The transcriptional level of *LDLR* gene is highly controlled by cellular sterol levels though the activation of *SREBP2*, in which an elevation in cellular sterol level suppresses *LDLR* gene transcription and in contrast, a depletion of cellular sterols leads to an elevated *LDLR* mRNA abundance and subsequently, an increased clearance of plasma LDL particles [67,68]. The gene expression data were consistent with the TC reduction in the cells treated with this honey (Figure 5B) and its high quantity and activity of antioxidants (72.1 mg/100 g), suggesting phenolics in manuka could confer the cholesterol-lowering effect through the activation of *AMPK, SREBP* and by upregulating the transcription of its downstream *LDLR* and *LXRα* genes, while maintaining the transcriptional level of *HCGMR* gene as in the control.

Arjuna honey (Figure 6C) at 2.0% induced *SREBP2* and highly upregulated *LDLR* gene expression (2.3-fold change relative to control) but did not upregulate *HMGCR* gene transcription. This honey, however, did not show its effect on the transcriptional levels of *LXRα* gene. These results are consistent with the TC reduction in HepG2 cells treated with this honey (Figure 5C), indicating that 2% arjuna honey are effective in lowering cholesterol through the activation of *AMPK-SREBP2* pathway. The findings are supported by previous studies demonstrating that *T. arjuna* extracts decreased plasma lipids and increased HDL to reduce the severity of atherosclerotic lesion in aorta [69] and modify lipid profile in human subjects with coronary artery disease [70].

Guggul honey (Figure 6D) activated both *AMPK* and *SREBP2* and it showed a stronger effect on the *SREBP2* gene at 2% concentration (2.2-fold change compared to control). This honey also induced *HCGMR* against the effect of the analogue and increased *LDLR* mRNA transcription at 1.0%, but not at 2.0% as expected. Instead, it upregulated *LXRα* gene expression at 2.0% concentration. Previous investigations indicated that an activation of *LXRα* leads to the upregulation of its direct targets and improves the transfer of cellular cholesterol to small high-density lipoproteins to prevent the formation of foam cells [71,72]. Thus, guggul honey through the activation of *LXRα* likely increased the transcriptional levels of transporter genes and proteins, leading to a TC reduction as observed in Figure 5D. Based on the responsive trend of the genes, we hypothesize that clearer effects of guggul honey on the tested genes could be observed at a later terminating time of the experiment. However, further study should include transporter genes and protein analysis to better understand the effect of guggul honey on hypercholesterolemia.

Jiaogulan honey (Figure 6E) induced the expression of the tested genes in a pattern that should lead to a lower TC content, but this TC reduction was not recorded (Figure 5E). Our TC data obtained for this honey opposed previous studies, which reported that jiaogulan extracts (0.1–0.3 mg/mL) reduce TC in primary hepatocytes [73] and total saponin extract of jiaogulan leaves activates AMPK, LXRα and its target transporters (ABCG5 and ABCG8) and reduces TC in hepatocytes of animal models [27]. Similarly, olive honey (Figure 6F) stimulated *SREBP2*, *LDLR* and *LXRα* genes at 2.0% concentration which could be relevant for the TC reduction, but this expectation also did not happen (Figure 5F). As olive retentate contains an enriched and purified source of low-molecular-weight polyphenols that are usable for food and pharmaceutical industries, it is supposed to confer a protective effect on oxidative stress and cholesterol homeostasis [74]. However, it is evidenced that cholesterol exits as free cholesterol and cholesteryl esters in hepatocytes [75]. In the current investigation, although jiaogulan and olive honeys did not lead to a decrease in TC content after 24 h (Figure 5E,F), they possibly induced a conversion of free cholesterol to cholesteryl esters through the esterification of cholesterol with fatty acids to protect the cells from free cholesterol toxicity, while modulating the tested genes in the pattern of *AMPK-SREBP2* pathway, responsive to a reduction in cellular free cholesterol content [64].

The honeys were also tested for their effect on *PPARα* gene, a central molecule in lipid catabolism. Manuka and guggul honeys upregulated *PPARα* gene expression at both 1.0% and 2.0% (*w*/*v*), whereas arjuna and jiaogulan honeys increased this gene transcription at 2.0% (Figure 6A–E). However, the gene expression results did not correspond to the high TG content in HepG2 cells treated with the honeys. Although it was reported that the activation of *PPARα* suppresses the availability of triglycerides for the assembly of very low-density lipoprotein and increases hepatic TG accumulation [76], the mechanism through which the tested honeys activated *PPARα*, its direct targets and the links to the increased triglyceride accumulation in HepG2 cells remain unclear.

## 4. Conclusions

The current study demonstrated that the quality of manuka and four newly developed honeys (arjuna, guggul, jiaogulan and olive), in terms of physicochemical properties, comply with the international quality regulations (EEC, 110/2001), excluding olive honey which contains a low monosaccharide content (49.2%). FTIR analysis of the honeys confirmed the presence of the functional groups which are typical for natural honeys. Arjuna and guggul honeys showed their highest phenolic content (73.4 and 108.2 mg GAE/100 g, respectively), correlating with their strongest RSA (up to 90.8% and 91.7%) and an outstanding AEAC (81.0 and 81.9 mg/100 g honey). Manuka honey also contains a high amount of phenolics as arjuna does (72.1 mg GAE/100 g) but its flavonoids are relatively low (3.9 mg CAE/100 g) and it elicited a moderate RSA (55.5%) and AOAC (49.6 mg/100 g). Olive honey demonstrated the highest quantity of flavonoids (24.0 mg CAE/100 g), a significant amount of phenolics (59.8 mg GAE/100 g), a considerable RAS (75.2%) and AOAC (67.1 mg/100 g). Jiaogulan honey exhibited the lowest antioxidant quantities and activities but it was the most cytotoxic of the tested honeys. Particularly, 5% and 10% of this honey reduced cell viability up to 93.5% and 92.0% after 72 h and 24 h, respectively.

Only manuka and jiaogulan honeys activated *Nrf2*, but all the tested honeys upregulated the downstream *NQO1* gene expression and they showed a stronger effect on this gene at 2.0% concentration (Figure 4). Preliminary results for the protective effects of the tested honeys on cholesterol homeostasis were obtained. Manuka (1.0% and 2.0%), arjuna (2.0%) and guggul show a good agreement in terms of the reduced TC and the transcriptional level of the tested genes. Jaogulan and olive modulated the tested gene in the pattern that should lead to a TC reduction, but this reduction did not occur (Figure 5E,F). They possibly protected the cells from increased cholesterol accumulation that we recorded through stimulating the conversion of free cholesterol to cholesteryl esters, while they modulated the tested genes in the pattern of *AMPK-SREBP2* pathway to reduce cellular free cholesterol content [64]. Although the tested honeys (except olive honey) activated *PPARα*, all of them did not reduce cellular triglyceride content in fatty acid-induced HepG2 cells. For the first time the current study provides evidence on the physicochemical quality, antioxidant properties and the protective effects of manuka and four newly developed honeys (arjuna, guggul, jiaogulan and olive) on cholesterol homeostasis at cellular and molecular levels, however, further investigations are needed to better understand their mode of action in lipogenesis.

## Figures and Tables

**Table 1:** Composition of medicinal nectars.

Components	Concentration	Reference
Sugars (sucrose:hexoses)	50.0% (3:1, *w*/*w*)	[21,22]
Amino acids	2.4 mM	[23]
Plant extracts	<1.0% (*w*/*w*)	[8,28]

**Table 2:** Physicochemical properties of honeys.

Parameters	MH1	ARJ	GUG	JIA	OLI
Moisture content (%)	18.5 ± 0.8 ^c^	14.9 ± 0.6 ^b^	13.4 ± 0.2 ^a^	14.0 ± 0.2 ^ab^	18.4 ± 0.2 ^c^
Electrical conductivity (mS/cm)	0.54 ± 0.01 ^c^	0.24 ± 0.01 ^a^	0.34 ± 0.01 ^b^	0.31 ± 0.01 ^b^	0.32 ± 0.01 ^b^
pH	4.1 ± 0.05 ^ab^	3.9 ± 0.05 ^a^	4.0 ± 0.01 ^ab^	4.1 ± 0.01 ^ab^	4.2 ± 0.01 ^b^
Color intensity (mAU)	2201.1 ± 53 ^e^	462.3 ± 16 ^c^	722.7 ± 15 ^d^	171.7 ± 5 ^a^	243.5 ± 12 ^b^

Data are mean ± SD of four replicate measurements with different letters in the same row indicating significant differences (*p* < 0.05), analyzed by one-way ANOVA succeeded by Duncan’s multiple comparison test. MH1: manuka honey containing methylglyoxal 400 mg/kg, ARJ: arjuna honey, GUG: guggul honey, JIA: jiaogulan honey and OLI: olive honey. mS: milli-siemens, mAU: milli-absorbance unit.

**Table 3:** Biochemical characteristics of medicinal honey.

Parameters	MH1	ARJ	GUG	JIA	OLI
Fructose (g/100 g)	44.2 ± 2.0 ^e^	33.5 ± 0.3 ^b^	37.6 ± 0.4 ^c^	41.4 ± 0.9 ^d^	28.0 ± 2.8 ^a^
Glucose (g/100 g)	28.9 ± 2.0 ^b^	27.4 ± 0.1 ^b^	31.9 ± 0.2 ^c^	33.7 ± 0.3 ^c^	21.2 ± 2.3 ^a^
Protein content (mg/100 g)	128.5 ± 1.0 ^e^	25.6 ± 5.0 ^a^	55.3 ± 3.0 ^b^	111.4 ± 3.6 ^d^	66.6 ± 9.1 ^c^
Total phenolics (mg GAE/100 g)	72.1 ± 2.0 ^c^	73.4 ± 2.4 ^c^	108.2 ± 1.8 ^d^	31.6 ± 1.7 ^a^	59.8 ± 0.5 ^b^
Total flavonoids (mg CAE/100 g)	3.9 ± 0.2 ^b^	15.3 ± 0.4 ^c^	4.1 ± 0.5 ^b^	2.1 ± 0.1 ^a^	24.0 ± 0.5 ^d^
Radical scavenging activity (%)	55.5 ± 1.7 ^b^	90.8 ± 0.4 ^d^	91.7 ± 0.4 ^d^	21.7 ± 0.3 ^a^	75.2 ± 0.5 ^c^
AEAC (mg/100 g)	49.6 ± 1.5 ^b^	81.0 ± 0.3 ^d^	81.9 ± 0.4 ^d^	19.4 ± 0.4 ^a^	67.1 ± 0.3 ^c^

Data are mean ± SD of four replicate measurements, with different letters in the same row (a–e) indicating significant differences (*p* < 0.05) by one-way ANOVA followed by Duncan’s multiple-range test. MH1: manuka honey containing methylglyoxal 400^+^ mg/kg, ARJ: arjuna honey, GUG: guggul honey, JIA: jiaogulan honey and OLI: olive honey. GAE: gallic acid equivalent, CAE: catechin equivalent, AEAC: ascorbic acid equivalent antioxidant content.

## Data Availability

Data Availabile on request.

## Supplementary Materials

The following are available online at https://www.mdpi.com/2072-6643/13/1/151/s1, Table S1: Primer sequences used in real time qPCR.
