# Supplementary material for: Physicochemical Properties and Effects of Honeys on Key Biomarkers of Oxidative Stress and Cholesterol Homeostasis in HepG2 Cells
Source: Nutrients. 2021 Jan 5;13(1):151. doi: 10.3390/nu13010151 (PMC7824776; doi:10.3390/nu13010151)
Supplement: Supplementary file 1 [file nutrients-13-00151-s001.pdf]

Table S1: Primer sequences used in real time qPCR.

Gene	RefSeq Identification	Primers	Sequence (5' to 3')
AMPKα	NM_006252.4	Forward	TCGCCACTCTCCTGATGCATAT
		Reverse	GATGATGAGGCTGTGAAAGAAG
Nrf2	NM_001145413	Forward	GAGCCCAGTATCAGCAACAG
		Reverse	TTCAATGATTCTGACTCCGGC
NQO1	NM_001025434	Forward	TGCTGCAGCGGCTTTGAAGA
		Reverse	TTTCAGTATCCTGCCGAGTCT
SREBP2	NM_004599.4	Forward	GCCCTGGAAGTGACAGAGAG
		Reverse	TCACTCCCTGGGAAAGCA
HMCGR	NM_000859.3	Forward	GGTGTATCTATTCGCCGACAG
		Reverse	CTGTTGGAGTGG CAGGACC
LDLR	NM_000527.4	Forward	CATCTACTCGCTGGTGACTG
		Reverse	GGCAACCGGAAGACCATCTT
LXRα	NM_001130102.3	Forward	TCACCTTCCTCAAGGATTTCA
		Reverse	TCATCAACCCCATCTTCGAG
SREBP-1c	NM_001321096.3	Forward	CAGCTCTGCACTCCTTCAAG
		Reverse	TGCAGCTGTTCCTGTGTGAC
PPARα	NM_001001928	Forward	CAATGCACTGGAACTGGATGA
		Reverse	AGACTCCACCTGCAGAGCAA
β-actin	NM_001101.5	Forward	GGTCAGAAGGATTCCTATGTGG
		Reverse	GCACCACACCTTCTACAATGAG
